# Glu-108 in Saccharomyces cerevisiae Rad51 Is Critical for DNA Damage-Induced Nuclear Function

**DOI:** 10.1128/mSphere.00082-19

**Published:** 2019-03-20

**Authors:** Tanvi Suhane, Vijayalakshmi Bindumadhavan, Nupur Fangaria, Achuthsankar S. Nair, Wahida Tabassum, Poorvaja Muley, Mrinal K. Bhattacharyya, Sunanda Bhattacharyya

**Affiliations:** a Department of Biotechnology and Bioinformatics, School of Life Sciences, University of Hyderabad, Hyderabad, India; b Department of Computational Biology and Bioinformatics, University of Kerala, Thiruvanthapuram, India; c Department of Biochemistry, School of Life Sciences, University of Hyderabad, Hyderabad, India; Carnegie Mellon University

**Keywords:** DNA repair, Hsp90, homologous recombination, Hsp90-Rad51 interaction, Rad51 recruitment to chromatin, molecular chaperone

## Abstract

DNA damage-induced Rad51 focus formation is the hallmark of homologous recombination-mediated DNA repair. Earlier, we reported that Rad51 physically interacts with Hsp90, and under the condition of Hsp90 inhibition, it undergoes proteasomal degradation. Here, we show that the dynamic interaction between Rad51 and Hsp90 is crucial for the DNA damage-induced nuclear function of Rad51. Guided by a bioinformatics study, we generated a single mutant of Rad51, which resides at the N-terminal domain, outside the ATPase core domain. The mutant with an E to L change at residue 108 (Rad51^E108L^) was predicted to bind more strongly with Hsp90 than the wild-type (Rad51^WT^). A coimmunoprecipitation study demonstrated that there exists a distinct difference between the *in vivo* associations of Rad51^WT^-Hsp90 and of Rad51^E108L^-Hsp90. We found that upon DNA damage, the association between Rad51^WT^ and Hsp90 was significantly reduced compared to that in the undamaged condition. However, the mutant Rad51^E108L^ remained tightly associated with Hsp90 even after DNA damage. Consequently, the recruitment of Rad51^E108L^ to the double-stranded broken ends was reduced significantly. The *E108L*-*rad51* strain manifested severe sensitivity toward methyl methanesulfonate (MMS) and a complete loss of gene conversion efficiency, a phenotype similar to that of the *Δrad51* strain. Previously, some of the N-terminal domain mutants of Rad51 were identified in a screen for a Rad51 interaction-deficient mutant; however, our study shows that Rad51^E108L^ is not defective either in the self-interaction or its interaction with the members of the Rad52 epistatic group. Our study thus identifies a novel mutant of Rad51 which, owing to its greater association with Hsp90, exhibits a severe DNA repair defect.

**IMPORTANCE** Rad51-mediated homologous recombination is the major mechanism for repairing DNA double-strand break (DSB) repair in cancer cells. Thus, regulating Rad51 activity could be an attractive target. The sequential assembly and disassembly of Rad51 to the broken DNA ends depend on reversible protein-protein interactions. Here, we discovered that a dynamic interaction with molecular chaperone Hsp90 is one such regulatory event that governs the recruitment of Rad51 onto the damaged DNA. We uncovered that Rad51 associates with Hsp90, and upon DNA damage, this complex dissociates to facilitate the loading of Rad51 onto broken DNA. In a mutant where such dissociation is incomplete, the occupancy of Rad51 at the broken DNA is partial, which results in inefficient DNA repair. Thus, it is reasonable to propose that any small molecule that may alter the dynamics of the Rad51-Hsp90 interaction is likely to impact DSB repair in cancer cells.

## Abstract (precis)

Rad51-mediated homologous recombination is the major mechanism for repairing DNA double-strand break (DSB) repair in cancer cells. Thus, regulating Rad51 activity could be an attractive target. The sequential assembly and disassembly of Rad51 to the broken DNA ends depend on reversible protein-protein interactions. Here, we discovered that a dynamic interaction with molecular chaperone Hsp90 is one such regulatory event that governs the recruitment of Rad51 onto the damaged DNA. We uncovered that Rad51 associates with Hsp90, and upon DNA damage, this complex dissociates to facilitate the loading of Rad51 onto broken DNA. In a mutant where such dissociation is incomplete, the occupancy of Rad51 at the broken DNA is partial, which results in inefficient DNA repair. Thus, it is reasonable to propose that any small molecule that may alter the dynamics of the Rad51-Hsp90 interaction is likely to impact DSB repair in cancer cells.

## INTRODUCTION

Whenever cells are exposed to DNA-damaging agents, the family of DNA repair proteins must relocate to the nucleus and be recruited to the damaged chromatins to elicit a DNA damage response and to ensure efficient repair of damaged DNA (1–3). These groups of proteins include DNA damage signaling proteins (Mre11, ATM, ATR, and DNA-PKcs), cell cycle checkpoint effectors (Chk1 and Chk2), and DNA processing enzymes (Mre11, ExoI, Sae2, Rad51, Rad52, Rad54, BRCA1/2, BLM, Ku70/80, ligase IV, etc.) (4). The sequential assembly and disassembly of DNA repair proteins at DNA broken ends depend on reversible protein-protein interactions. Rad51, a central player of homology-directed double-strand break (DSB) repair, remains in the cytoplasm under normal conditions. DNA damage leads to the redistribution of Rad51 from the cytoplasm to the nucleus and its loading onto the broken ends of DNA. It is reasonable to propose that insufficient recruitment of Rad51 onto the chromatin is likely to have a severe impact on homologous recombination (HR) efficiency. Earlier reports demonstrated that in a human cell line, BRCA1 promotes the localization of BRCA2 to damage foci through the BRCA2 binding protein PALB2 (5–8). BRCA2 interacts with RAD51 and promotes RAD51 assembly onto single-stranded DNA (ssDNA) (9–11). However, BRCA2 is absent in lower eukaryotes, where HR is the predominant pathway for DNA repair. It is reported that in Saccharomyces cerevisiae, Rad52 promotes Rad51 filament assembly (12) by interacting with RPA. Rad52 is thought to replace RPA bound to ssDNA with Rad51 or provide a seeding site within the RPA-bound ssDNA for subsequent binding of Rad51 (13).

Our previous study revealed that Rad51 is a direct client of Hsp90 and is dependent upon Hsp90 for its maturity and activity (14). Apart from merely providing maturity to the client proteins, Hsp90 also assists in the translocation of proteins to different cellular compartments (15). Previous reports have established that the Hsp90 chaperone machinery not only escorts steroid hormone receptors (SHRs) to the nucleus but is also responsible for the recycling of the receptor on chromatin and stabilizing the DNA-binding properties of the receptor (16). Two cochaperones of Hsp90, p23 and Bag-1L, are found to modulate steroid hormone receptor function by controlling receptor binding to chromatin (16).

Our earlier study demonstrated that the charged linker deletion mutant of yHsp90 (Δ*211-259hsp82*) inhibits effective Rad51 focus formation in the nucleus upon DNA damage (14). This finding was positively correlated with severe methyl methanesulfonate (MMS) sensitivity (comparable to that for the Δ*rad51* strain) and with the complete loss of Rad51-dependent gene targeting function. We demonstrated that the charged linker deletion (Δ*211-259hsp82*) mutant strain is strikingly different than the wild-type strain in the distribution of Rad51 foci upon MMS treatment. Although there was only a 20% overall reduction in the Rad51 focus formation, the number of nuclei having multiple foci was drastically reduced in the mutant strain. This clearly indicates that in mutant nuclei, effective Rad51 levels may be low. Since the charged linker region is responsible for providing structural flexibility between amino and carboxyl-terminal domains of Hsp90 (17), an optimum interaction between Rad51 and Hsp90 may be compromised in the mutant. Hence, we hypothesize that effective Hsp90 and Rad51 interaction may be crucial for nuclear function of Rad51. To prove this, we utilized a bioinformatics approach to design a point mutant with an E to L change at residue 108 (Rad51^E108L^), which has a stronger affinity toward Hsp90. Our data reveal that there exists a dynamic equilibrium between the association of wild-type Rad51 (Rad51^WT^) and Hsp90 under a normal condition and dissociation under DNA-damaging conditions. In the case of Rad51^E108L^, due to tighter association, the interaction between Hsp90 and mutant Rad51 becomes irreversible; hence, even under DNA-damaging conditions, the mutant Rad51 protein does not proficiently dissociate from Hsp90. As a result, the mutant Rad51^E108L^ is not recruited to the broken DNA ends as efficiently as wild-type Rad51. Hence, the *E108L-rad51* strain shows extreme sensitivity toward DNA-damaging agents and poor gene conversion activity. This study points out that the DNA damage-induced reversible protein-protein interaction between Rad51 and Hsp90 plays a critical role in Rad51 function.

## RESULTS

### Generation of *RAD51* mutant strain based on the molecular docking studies between yHsp90 and Rad51.

**FIG 1:**
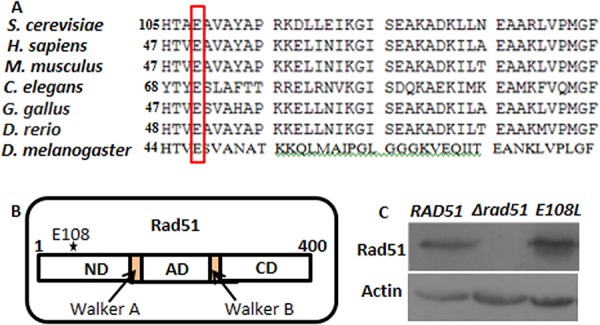
Generation of *RAD51* mutant strain. (A) Multiple sequence alignment of Rad51 (N-terminal domain) protein sequences of S. cerevisiae (yeast) with Homo sapiens (human), Mus musculus (mouse), Gallus gallus (bird), Danio rerio (zebrafish), Caenorhabditis elegans (nematode), and Drosophila melanogaster (fruit fly). The conserved glutamic acid residues among various organisms are represented by the red box. (B) Schematic representation of Rad51 domains demonstrating boundaries of N-terminal, ATPase (AD), and C-terminal domains along with the Walker A and Walker B motifs. The star depicts the approximate location of E108 in the N-terminal domain of Rad51. (C) Western blot was performed using protein extracts from wild-type, *Δrad51*, and *E108L-rad51* strains. Actin was used as a loading control.

Earlier studies in our lab demonstrated that yHsp90 and Rad51 can physically interact (14). Unlike other chaperones, there is no specific binding pocket present in Hsp90 through which it binds to the client proteins. Hence, in order to understand the point of contacts between yHsp90 and Rad51, we employed a bioinformatics approach. To that end, Rad51 proteins (PDB identifier [ID] 1SZP) having various combinations of monomers, dimers, and hexamers were allowed to dock with yHsp90 (PDB ID 2CG9) using the fully automated web-based program ClusPro 2.0 (18), which employs the improved fast Fourier transform (FFT)-based rigid docking program PIPER (19). Thirty models of the protein-protein complex for each type of interaction, namely, balanced, electrostatic favored, hydrophobic favored, and van der Waal's plus electrostatic, were generated for each docking. It was found that a hydrophobic-favored interaction showed the lowest energy scores; hence, the corresponding protein complex model with the largest cluster was chosen. The surface view of the three-dimensional structure of Rad51 displays a characteristic pocket in each of the monomers into which the yHsp90 is found to dock. The docked complex models showed that the N-terminal residue of the Rad51 E chain, Glu 108 (1.88 Å), has the shortest bond distance with yHsp90 C-terminal residues. We conducted a multiple-sequence alignment of Rad51 (Fig. 1A) and found that E108, which is predicted to have the strongest association with Hsp90, is evolutionarily conserved. In Rad51, the amino acid residue E108 is present in the N-terminal domain of Rad51, which lies outside its catalytic domain (Fig. 1B). To explore whether the Hsp90 and Rad51 association mediates Rad51 nuclear function under DNA-damaging conditions, one approach may be the generation of a Rad51 mutant with a reduced affinity for Hsp90. However, as Rad51 is a client of Hsp90, we reasoned that any mutant of Rad51 that fails to interact with Hsp90 due to a low affinity would be unstable in the cell. Hence, we designed a strong-affinity mutant to establish our hypothesis. By *in silico* mutation, we created four single mutants of Rad51 where the glutamic acid at the 108th position was replaced by neutral residues (glycine, alanine, leucine, and isoleucine). Table 1 displays a comparison of the parameters of yHsp90 docking with the wild-type and mutant Rad51 based on ClusPro results. Our study shows that the mutant Rad51^E108L^ and Hsp90 docked complex results in a maximum increase in cluster size of 139 compared to 71 for the wild type. This implies a greater probability of the receptor-ligand complex being found in that specific conformation. Furthermore, there is a decrease in the energy score from −1,407.2 to −1,512.6 between the wild-type and Rad51^E108L^ mutant, respectively, which points to an increased stability of the protein complex. The *rad51* mutant was subsequently cloned into a yeast 2µ expression vector pTA (20) having the GPD promoter. As the Rad51 and Hsp90 interaction is essential for the stability of Rad51, we determined the stability of Rad51 mutant proteins by Western blot analysis. For this, we generated yeast strains NRY1, NRY2, and TSY17 by transforming empty vector (pTA), pTA-*RAD51*, and pTA*-E108L-rad51* vectors into a null *rad51* yeast strain. The steady-state level of the mutant Rad51 was comparable to that of the wild type (Fig. 1C).

**TABLE 1:** ClusPro results depicting cluster sizes and energy scores of yHsp90 (2CG9A) with wild-type and mutant Rad51

Rad51 (1SZP ABCDEF) strain	Hydrophobic-favored interaction	
	Cluster size	Energy score
Wild-type (E108)	71	−1,407.2
E108G	117	−1,534.0
E108A	117	−1,518.4
E108I	113	−1,543.3
E108L	139	−1,512.6

### Rad51^E108L^ shows a stronger association with Hsp90 than the wild-type Rad51.

**FIG 2:**
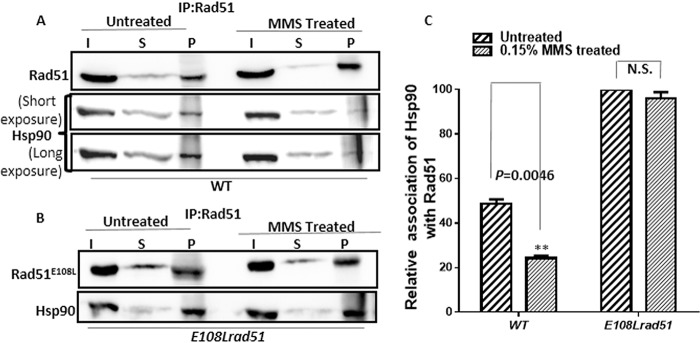
Rad51^E108L^ shows a stronger association with Hsp90 than the wild-type Rad51. (A) Western blot showing coimmunoprecipitation of Rad51 with Hsp90 from whole-cell extracts of wild-type strain and cells treated with 0.15% MMS for 2 h. I, input; S, supernatant; P, pellet. (B) Western blot showing coimmunoprecipitation of Rad51^E108L^ with Hsp90 from whole-cell extracts of *E108L-rad51* mutant strain untreated and treated with 0.15% MMS for 2 h. Immunoprecipitation (IP) was performed using an anti-Rad51 antibody. An anti-Hsp90 antibody was used for Western blotting. (C) Relative association of Hsp90 with Rad51 was calculated from at least three independent experiments, and standard deviations are plotted for both wild-type and mutant strains. *P* values were calculated using the two-tailed Student’s *t* test. **, *P = *0.0046; N.S., not significant.

To investigate the interaction between Rad51 and yHsp90, we performed coimmunoprecipitation experiments under normal as well as MMS treatment conditions. To capture a detectable association between yHsp90 and Rad51, we overexpressed both y*HSP90* and *RAD51* (or its mutant version) from two 2µ vectors, each having a GPD promoter. The yHsp90-Rad51 complex was coimmunoprecipitated from the whole-cell extract with an anti-Rad51 antibody, followed by detection on a Western blot using an anti-Hsp82 antibody (Fig. 2A and B). Under normal conditions, in the wild-type strain, a small fraction of Hsp90 was associated with Rad51, whereas, in the case of the mutant strain, a significantly larger fraction of Hsp90 was associated with Rad51. Quantification of the several experimental repeats showed that the relative association between Hsp90 and Rad51^E108L^ was almost double the association found between Hsp90 and Rad51^WT^. This signifies a stronger association of Hsp90 with Rad51^E108L^ than with Rad51^WT^. In the presence of MMS, Hsp90 and Rad51 association was reduced in the wild-type strain. On the other hand, in the *E108L-rad51* strain, even in the presence of MMS, there was no detectable reduction in the association between Hsp90 and Rad51^E108L^. We repeated this experiment three times and calculated the relative association of Hsp90 with Rad51 in the presence and absence of MMS. Our analysis shows that approximately 50% dissociation of the Rad51^WT^-Hsp90 complex occurs upon MMS treatment, whereas no significant dissociation of the Rad51^E108L^-Hsp90 complex was observed under similar conditions (Fig. 2C). Thus, from this experiment, we conclude that there is a dynamic equilibrium between Rad51-Hsp90 complexes: in the presence of DNA damage, the equilibrium is shifted toward the dissociation of Rad51-Hsp90. However, this dynamic interaction is absent in the *E108L-rad51* strain, and the complex remains in the associated form even in the presence of the DNA-damaging agent.

### *HO*-induced Rad51 recruitment to the broken DNA ends is compromised in the *E108L-rad51* strain.

**FIG 3:**
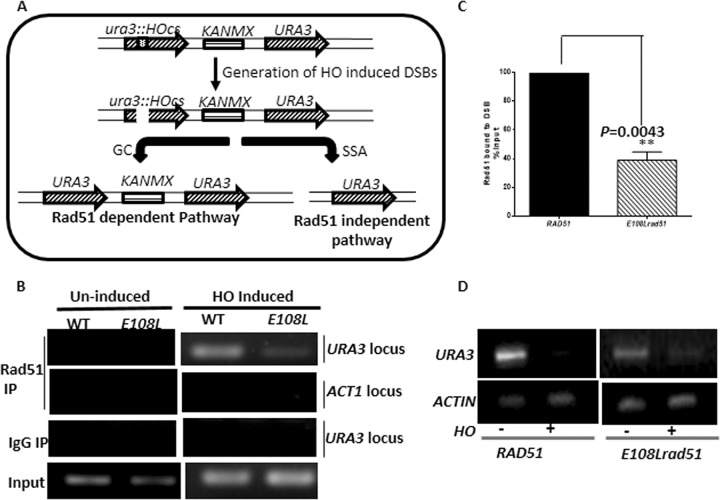
*HO*-induced Rad51 recruitment to the broken DNA ends is severely compromised in *E108L-rad51* mutant. (A) Schematic diagram of a cassette incorporated in the strain used for studying gene conversion efficiency. It harbors two copies of *URA3*, one of which is mutated by the insertion of an *HO* endonuclease site. Induction with galactose creates single DSB in the mutated *ura3*, repair of which takes place in either a Rad51-dependent or Rad51-independent manner. *KANMX* cassette will be retained only if repair happens via the Rad51-dependent manner. (B) Chromatin immunoprecipitation (ChIP) of strains expressing wild-type Rad51 and *E108L-rad51*. Gel image showing one of the representative PCR products of input and precipitated samples using *URA3* donor-specific primer and *ACT1*-specific primer. Immunoprecipitation was performed using anti-Rad51 and IgG antibodies. Input represents the total amount of DNA in the sample. (C) Each set was repeated three times, and the band intensities of the recruited samples upon HO induction were quantified using ImageJ software; comparative recruitment of Rad51 and Rad51^E108L^ is plotted with respect to the input. Error bars indicate standard deviations (SDs); *n* = 3 (*P* values were calculated using the two-tailed Student's *t* test). **, *P < *0.01. (D) Semiquantitative reverse transcriptase PCR (RT-PCR), representing the amplification of DNA around the DSB site in *ura3* before and after HO endonuclease induction. Lower intensity of band in HO-induced sample indicates the DSB generation in strains having wild-type Rad51 and *E108L-rad51*. Actin was used as a loading control.

During homologous recombination-mediated DNA repair, Rad51 is recruited to the ssDNA overhangs. It searches for the homologous DNA and, once found, facilitates the repair by performing a strand exchange reaction. The recruitment of Rad51 to the broken ends is the hallmark of DNA repair. Our previous observations suggest that the *E108L-rad51* mutant is defective in dissociating from Hsp90 upon DNA damage. This defect may cause inadequate recruitment of Rad51 mutants to the broken DNA. To study the recruitment of mutant Rad51 to the DSB, we employed chromatin immunoprecipitation (ChIP) assays. To that end, we used NA14 strains (21) harboring null *rad51*. We modified the NA14 strain and generated three strains, namely, TSY20, TSY21, and TSY22, where native *RAD51* is knocked out, and into those backgrounds, the empty plasmid, wild-type *RAD51*, and the mutant *rad51* were transformed, respectively. These strains have a cassette inserted in chromosome V with two copies of *URA3*, separated by 3 kb, of which one *ura3* copy is inactivated by the insertion of an *HO* endonuclease restriction site (Fig. 3A). The *KANMX* gene is incorporated within the two *URA3* genes. *HO* endonuclease is expressed in the strain by a galactose inducible promoter. A double-strand break (DSB) is generated in the *ura3* gene upon induction of *HO* endonuclease. We pulled down the Rad51-bound DNA segments from uninduced and *HO*-induced samples and subsequently compared the recruitment of mutant Rad51 protein to the donor *URA3* locus (22). This experiment was repeated three times, and representative data from one of these are presented (Fig. 3B). To ensure the specificity of Rad51 recruitment to the broken locus, we probed its recruitment at the *ACT1* locus, which does not contain an *HO* cut site. We did not detect any band at the *ACT1* locus. We quantified the extent of recruitment of Rad51 proteins by measuring the ratio of amplification in the pellet sample with respect to the amplification observed in the input. To confirm the specificity of Rad51 recruitment to the DSB, we performed ChIP with IgG, which does not result in any amplification with the precipitated sample (Fig. 3B). Although there was no recruitment of Rad51 in the *HO*-uninduced condition, upon *HO* induction, the recruitment of Rad51^E108L^ was only 40% of that for the wild type (Fig. 3C). To ensure that the defect in the recruitment of the mutant Rad51 to the DSB was not due to the inefficiency of galactose-induced DSB, we probed the *HO* endonuclease recognition site in the presence and absence of *HO* induction. To that end, we amplified the *HO* site flanking the *ura3* region using a forward primer, which is 20 bp upstream of the *HO* site, and a reverse primer, which is complementary to the middle part of *KANMX* gene. We observed the amplification of the target region in a galactose-untreated sample; however, after 1 h of galactose induction, the amplicon disappeared, indicating the successful generation of DSBs in all the strains (Fig. 3D). Overall, from these experiments, we conclude that the effective concentration of the Rad51^E108L^ mutant at broken DNA ends is less than that of the wild-type Rad51.

### Mutation at the E108 position of Rad51 sensitizes the cells to MMS and renders them deficient in gene conversion.

**FIG 4:**
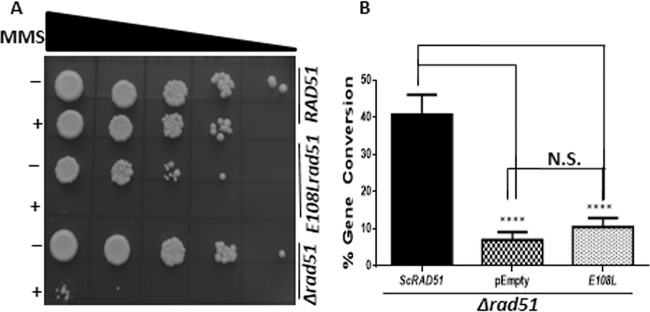
Mutation at E108 position of Rad51 sensitizes the cells to MMS and renders them deficient in gene conversion. (A) Pictorial representation of return-to-growth assay upon MMS treatment. Cells were spotted after serial dilution of treated and untreated cells for wild-type and mutant strains. First lane for each strain shows untreated and second lane shows treated cells. (B) Graph showing the percentages of gene conversion. Cells were spread on galactose-containing plates and subsequently obtained colonies were patched on G418 sulfate plates. Percentage was determined by calculating the number of colonies grown on G418 sulfate plate versus number of colonies obtained on galactose plate. Error bars indicate SDs; *n* = 3; *P* values were calculated using the two-tailed Student’s *t* test. ****, *P < *0.0001; N.S., not significant.

In *S. cerevisiae*, homologous recombination is the preferred pathway for repairing DSBs, in which Rad51 plays a central role. To understand the effect of *rad51* mutation, we performed the return-to-growth assay upon DNA damage. This was conducted by exposing the strains to 0.03% MMS (methyl methanesulfonate) for 2 h. Subsequently, treated and untreated cells were serially diluted by 10-fold as presented in Fig. 4A and spotted on selective medium. We observed that the *E108L-rad51* strain showed a slow growth phenotype compared to that of the wild type and Δ*rad51* strains. The survivability of the cells was positively correlated with the efficiency of DNA repair. We observed that *E108L-rad51* cells were highly sensitive to MMS-induced DNA damage, similar to that observed in Δ*rad51* cells. The mechanism of homologous recombination involves repairing the DSBs by utilizing a homologous sequence from the genome. If the genome contains repetitive sequences and a double-strand break is created in any one of the repeats, it can be repaired by gene conversion, which is Rad51 dependent. We examined the gene conversion efficiency of the Rad51 mutant in the yeast strain NA14 (21). The DSB can be repaired by either of the two HR pathways (gene conversion or single strand annealing), and the repair products are easily distinguishable. If repaired by the Rad51-dependent gene conversion pathway, the strain behaves as G418 sulfate resistant; if it is repaired by the Rad51-independent single-strand annealing (SSA) pathway, the strain will be G418 sulfate sensitive (Fig. 3A) (21). The percent gene conversion was scored by growing cells on G418 sulfate-containing plates after galactose induction. Our experimental data indicate that there was no significant change in the gene conversion (GC) efficiency of the wild type (near 40%). However, the GC score for the *E108L-rad51* mutant (10.5%) was comparable to that of the Δ*rad51* strain (7%) (Fig. 4B). Overall, we conclude from our experimental data that the *E108L-rad51* mutant behaved as a complete loss-of-function mutant of Rad51 in our assay.

### Rad51^E108L^ can form homodimers and interacts efficiently with the Rad52 epistasis group of proteins.

**FIG 5:**
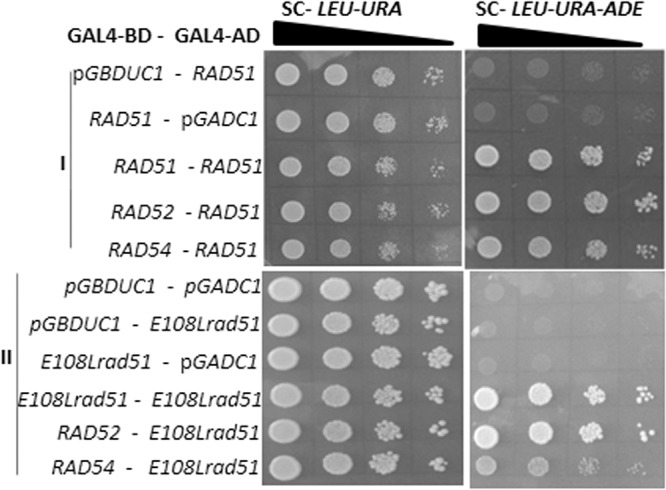
Rad51^E108L^ can form homodimers and bind efficiently to the Rad52 epistasis group of proteins. Yeast two-hybrid analysis depicting the interaction of *RAD51/rad51* mutants with Rad52 epistasis group. Various strains harboring bait and prey vectors are represented on the left. Cells of each strain were grown to an OD_600_ of 0.5 and serially diluted before spotting. To monitor the interaction between proteins, diluted cells were spotted on medium lacking Leu and Ura (left panel) as well as on medium lacking Leu, Ura, and Ade. Homodimerization as well as interaction of Rad51 (positive control) (I) and Rad51^E108L^ (II) with Rad52 and Rad54 was unaltered.

It has been established that to execute the nuclear function, Rad51 interacts with itself. Also, Rad52 and Rad54 modulate the catalytic activity of Rad51 via direct physical interaction. We wanted to test whether Rad51^E108L^ has any defect in self-association or association with Rad52 and Rad54. To that end, we used a yeast two-hybrid assay to measure the protein-protein interaction between Rad51^E108L^ and the Rad52 epistasis group. Figure 5 (top) shows the results with wild-type Rad51, which acts as a positive control in our study. The bottom of Fig. 5 shows that Rad51^E108L^ interacted efficiently with itself as well as with Rad52 and Rad54. We verified that Rad52 and Rad54 do not cause self-activation of a reporter gene (data not shown). No growth in a triple-drop-out plate for the strains PMY11 and PMY14 indicated that there was no self-activation for the indicative strains.

## DISCUSSION

Rad51 protein, which facilitates homologous strand exchange, is the central player for HR in mammalian cells. Disruption of this gene is associated with embryonic lethality in mice (23). It is reported that haploinsufficiency of this gene is linked with defects in human neurodevelopment (24, 25). The Rad51 focus formation in response to DNA damage is one of the regulatory events in HR.

Previously, we established that besides providing stability to the Rad51 protein, Hsp90 also controls its nuclear function, i.e., DNA damage-induced focus formation. Taking that study further, we show that the dynamic interaction between Hsp90 and Rad51 can influence the nuclear function of Rad51. We are reporting for the first time that DNA damage triggers the dissociation of Rad51 and Hsp90, which could be a prerequisite for the nuclear function of Rad51. Due to a stronger association with Hsp90, the Rad51^E108L^ protein probably remains locked with Hsp90; hence, the recruitment of Rad51^E108L^ to the broken DNA ends, even at a very high MMS concentration (0.15%), is considerably defective. This is evident by 10.5% GC efficiency and complete loss of cell survivability in the *E108L-rad51* mutant cells under DNA-damaging conditions. Thus, our study shows that there is a positive correlation between the extent of Hsp90-Rad51 dissociation after DNA damage and Rad51 nuclear activity. It appears that in the case of *E108L-rad51*, a major portion of the Hsp90 pool is associated with Rad51, which might result in an insufficient availability of free Hsp90 for other cellular functions. This is supported by our observation that the *E108L-rad51* mutant strain showed a slow growth phenotype compared to that of the wild-type strain. However, it is possible that the constitutive form of yHsp90, namely, Hsc82, might be sufficient for the essential cellular function of Hsp90, ensuring the survivability of the mutant strain.

A defect in recruitment to the damaged DNA may result from a defect in DNA binding or defects in its interactions with other nuclear proteins. An earlier report showed that glycine at the 103rd position of Rad51 is crucial for DNA binding (26). Another report showed that valine at 328, proline at 339, and isoleucine at 345 are also involved in DNA binding (27). Although there is no report available regarding the DNA binding capacity of the mutant used in our study, we do not anticipate any defect in DNA binding, as the mutant was recruited to the chromatin DNA albeit at lesser extent, probably due to the lesser availability of free Rad51^E108L^ proteins. In the case of the Rad51^E108L^ mutant, despite its apparent defect in reversible dissociation from Hsp90 under DNA-damaging conditions, its 40% recruitment confirms that it is not defective in DNA binding.

In our study, we expressed *RAD51* and *E108L-rad51* from episomal plasmids in a Δ*rad51* background and compared their phenotypes. Thus, it is important to ensure that the observed phenotypes were not due to overexpression. In an earlier study, it was observed that overexpression of Rad51 does not have any effect on MMS sensitivity or repair of a single DSB in wild-type cells. However, it sensitizes Δ*srs2* and Δ*ku70* strains toward MMS (28). It was also observed that a high level of Rad51 reduces the frequency of but does not eliminate HR (28). In our study, the steady-state levels of Rad51^WT^ and Rad51^E108L^ were comparable. Thus, the severe DNA repair defects observed in the *E108L-rad51* strain compared to that in *RAD51* cells are not due to overexpression but rather to the point mutation.

It did not escape our notice that nearly 50% less recruitment of Rad51 in the *E108L-rad51* strain had a profound effect on DNA repair. It is not unexpected, as our earlier study demonstrated that an only 20% reduction of Rad51 focus formation in the Δ*211-259hsp82* strain led to severe sensitivity to MMS and UV treatment (14). These findings prompted us to conclude that 20% to 50% less occupancy of Rad51 at the broken DNA ends is sufficient to perturb DSB repair.

The E108 residue of Rad51 that is in close proximity to Hsp90 resides outside the ATPase domain of Rad51 and is evolutionary conserved. The N-terminal domain of Rad51 is implicated in the monomer-monomer interaction as well as the interactions with the members of the Rad52 epistasis group (27, 29). Although the mutation is present in the N-terminal domain, it was not previously identified in Rad51 interaction-deficient mutants (30). The yeast two-hybrid assay confirms that the ability of Rad51^E108L^ for self-association as well as for associations with Rad52 and Rad54 are comparable to that of wild-type Rad51. As Rad51 recruitment to the broken DNA ends is an upstream event, the defect will be dominant over any other defects. Thus, the drastic phenotype found in the *E108L-rad51* strain is likely to be one of the primary causes for the loss-of-function phenotype in the mutant strain.

It is known that Hsp90 shows a variable degree of association with its clients. Hsp90 clients such as kinases are primarily associated with Hsp90 through transient interactions, and once chaperoned, they are readily released from Hsp90 as functional proteins. On the other hand, clients such as steroid hormone receptors remain associated with Hsp90 to maintain their functional forms. Also, the extent of association between Hsp90 and its client can alter the cellular function of its client. For example, the single point mutations in the epidermal growth factor receptor (EGFR^L858R^) and B-Raf kinase (B-Raf^V600E^) promote tumor formation. It was observed that these point mutants have enhanced levels of association with Hsp90 compared to those of their wild-type counterparts (31, 32). While binding with its clients, Hsp90 exhibits specificity toward the hydrophobic residues of proteins (33). The incorporation of leucine at the 108th position of Rad51 increases the hydrophobic stretch on Rad51 (107 to 113 amino acids). We speculate that such an increase in hydrophobicity might result in a tighter binding between Hsp90 and mutant Rad51 protein.

Collectively, our work establishes the importance of Hsp90 in the HR pathway, where it appears to regulate the stability and functions of Rad51. Increasing lines of evidence suggest that the functions of several DNA repair proteins, such as BRCA1, BRCA2, Chk1, DNA-PKcs, FANCA, and the Mre11/Rad50/NBS, are likely to be dependent on Hsp90 (34). A recent report showed that overexpression of Hsp90 leads to genomic instability through a negative regulation of the checkpoint kinase *RAD53* (22). Our work along with these reports embarks on the relationship of Hsp90 with DNA repair. Currently, DNA repair along with the Hsp90 inhibitor is being targeted in many cancer studies. Understanding the detailed regulation of HR will be beneficial for further knowledge in the field.

There are many reports which show that in response to various signals, Hsp90/Hsp82 gets posttranslational modifications (PTMs), and such PTMs help the release of the client protein (35–37). Currently, it is not known whether such PTM of Hsp90 occurs due to MMS treatment and that causes the decrease in association between Rad51^WT^ and Hsp90. It is also unclear how the stronger association between Rad51^E108L^ and Hsp90 was not overcome during the DNA damage response (DDR). These questions are interesting but beyond the scope of this report, and future studies might unravel the mechanism underlying the dissociation of Rad51 from Hsp90 upon DNA damage.

## MATERIALS AND METHODS

### Plasmids.

The sequences of all the primers used in this paper are tabulated in Table 2. The *RAD51* mutant (*E108L-rad51*) was cloned in 2µ yeast expression vector pTA (20) between the BamH1 and Pst1 restriction sites to generate the pTA-*E108L-rad51* plasmid. pTA-*RAD51* was used as a positive control in our study (20). Full-length *RAD51* and *E108L-rad51* were subcloned into prey vector pGADC1 and bait vector pGBDUC1 from pTA-*RAD51* and pTA-*E108L-rad51*, respectively. Thus, the plasmids pGADC1*/RAD51*, pGBDUC1/*RAD51*, pGADC1/*E108L-rad51*, and pGBDUC1/*E108L-rad51* were generated. Full-length *RAD52* was amplified using the OSB330/OSB331 primer set and cloned into pGBDUC1 vector between EcoRI and SalI restriction sites to create the pGBDUC1/*RAD52* plasmid. To generate the pGBDUC1/*RAD54* plasmid, *RAD54* was amplified using the OSB332/OSB333 primer set and cloned into pGBDUC1 vector between EcoRI and SalI restriction sites.

**TABLE 2:** Primer list

Primer	Sequence (5′→3′)	Purpose
OMKB90	GGATCCATGTCTCAAGTTCAAGAAC	Forward primer to amplify full-length *RAD51*
OMKB88	CTGCAGCTACTCGTCTTCTTCTC	Reverse primer to amplify full-length *RAD51*
OMKB149	GTCGACCTCGTCTTCTTCTCTGG	Reverse primer used to clone *E108L-rad51* into pET22b vector
OSB305	CTCGGATCCATGTCTCAAGTTCAAGAACAAC	Forward primer used to amplify full-length *rad51* mutants
OSB293	GTCGTCGACCTCGTCTTCTTCTCTGGGG	Reverse primer used to amplify full-length *rad51* mutants
OSB315	AGTGGGCTTCACACTGCTTTGGCGGTAGCA	Forward primer to create *rad51* E108L mutation
OSB314	TCTGGGAGCATATGCTACCGCCAAAGCAGTG	Reverse primer to create *rad51* E108L mutation
OSB278	CATGCAAGGGCTCCCTAGC	Forward primer used to amplify *URA3* region for ChIP
OSB279	CAACCAATCGTAACCTTCATCT	Reverse primer used to amplify *URA3* region for ChIP
OSB289	GTTAGTTGAAGCATTAGGTCC	Forward primer used to confirm *HO* digestion
KanB1	TGTACGGGCGACAGTCACAT	Reverse primer used to confirm *HO* digestion
OSB21	GACGGATCCATGGCTAGTGAAACTTTTGAATTTC	Forward primer to amplify full-length *hsp82*
OSB22	CGGGTCGACCTAATCTACCTCTTCCATTTCGG	Reverse primer to amplify full-length *hsp82*
OSB16	TGACCAAACTACTTACAACTCC	Forward primer to amplify 307 bp of 3′ end of *ACT1*
OSB14	TTAGAAACACTTGTGGTGAACG	Reverse primer to amplify *ACT1*
OSB330	CATGAATTCATGAATGAAATTATGGATATCGATG	Forward primer to amplify *RAD52*
OSB331	CATGTCGACTCAAGTAGGCTTGCGTGCATG	Reverse primer to amplify *RAD52*
OSB332	CATGAATTCATGGCAAGACGCAGATTACC	Forward primer to amplify *RAD54*
OSB333	CATGTCGACTCAATGTGAAATATATTGAAATGC	Reverse primer to amplify *RAD54*

### Site-directed mutagenesis.

Point mutations were introduced in *RAD51* by using the splice overlap extension (SOE) PCR technique. A primer set was designed to incorporate the required mutation in *RAD51* at the desired location. Yeast genomic DNA was used as a template, and the full-length gene was amplified in two segments in order to insert the point mutation. For amplifying the first and second segments to generate the *E108L-rad51* mutation, primer sets OSB305/OSB314 and OSB315/OSB293 were used, respectively. Full-length *RAD51* containing the E108L mutation was then amplified by using the first two segments along with primer set OMKB90/OMKB88. The *rad51* mutant was then cloned into the pTA 2µ yeast expression vector using the sites BamH1 and PstI. After successful cloning, the pTA-*E108L-rad51* construct was sequenced to confirm the desired mutation. To create the *E108L-rad51* mutant, we changed the codon GAA to TTG.

### Yeast strains.

The strains used in this study are tabulated in Table 3. LS402 *Δrad51* was transformed with empty vector (pTA), pTA-*RAD51*, and pTA-*E108L-rad51* to generate NRY1, NRY2, and TSY17, respectively. For the gene conversion assay, pTA-*RAD51* and pTA-*E108L-rad51* were transformed into NA14 Δ*rad51* (21) to generate TSY21 and TSY22. For a negative control, the NA14 Δ*rad51* strain was transformed with pTA empty vector to generate TSY20. To perform the yeast two-hybrid analysis, empty pGADC1 and pGBDUC1 vectors were transformed into a pJ694a parent strain to generate the PMY3 yeast strain. To study the interaction of wild-type Rad51 with itself and with Rad52 and Rad54 proteins, PMY8, PMY9, and PMY10 were created by transforming prey*-RAD51* plus bait*-RAD51*, prey*-RAD51* plus bait*-RAD52*, and prey*-RAD51* plus bait*-RAD54* constructs, respectively, into the pJ694a strain. Similarly, to study the interaction of Rad51^E108L^ with itself and with Rad52 and Rad54, strains TSY10, PMY12, and PMY13 were generated by transforming prey*-E108L-rad51* plus bait*-E108L-rad51*, prey*-E108L-rad51* plus bait*-RAD52*, and prey*-E108L-rad51* plus bait*-RAD54* constructs, respectively. Strains PMY4, PMY7, PMY14, and PMY11 were utilized as controls. These strains were generated by transforming empty prey plus bait*-RAD51*, prey*-RAD51* plus empty bait, empty prey plus bait*-E108L-rad51*, and prey*-E108L-rad51* plus empty bait vectors, respectively, into the pJ694a strain.

**TABLE 3:** Yeast strains

Strain	Genotype	Source or reference
NRY1	*MAT***a** *leu2-3,112 trp1-1 can1-100 ura3-1 ade2-1 his3-11,15* [*phi^+^*] *RAD51*::*LEU2* pTA	20
NRY2	*MAT***a** *leu2-3,112 trp1-1 can1-100 ura3-1 ade2-1 his3-11,15* [*phi^+^*] *RAD51*::*LEU2* pTA*-RAD51*	20
TSY17	*MAT***a** *leu2-3,112 trp1-1 can1-100 ura3-1 ade2-1 his3-11,15* [*phi^+^*] *RAD51*::*LEU2* pTA*-E108L-rad51*	This study
TSY20	*MAT***a** *inc ura3-HOcs lys2*::*ura3-HOcs-inc ade3*::*GALHO ade2-1 leu2-3,112 his3-11,15 trp1-1 can1-100 RAD51*::*LEU2* pTA	This study
TSY21	*MAT***a** *inc ura3-HOcs lys2*::*ura3-HOcs-inc ade3*::*GALHO ade2-1 leu2-3,112 his3-11,15 trp1-1 can1-100 RAD51*::*LEU2* pTA-*RAD51*	This study
TSY22	*MAT***a** *inc ura3-HOcs lys2*::*ura3-HOcs-inc ade3*::*GALHO ade2-1 leu2-3,112 his3-11,15 trp1-1 can1-100 RAD51*::*LEU2* pTA-*E108L-rad51*	This study
PMY3	*MAT***a** *trpl-901 leu2-3,112 ura3-52 his3-200 ga14*Δ *ga180*Δ *LYS2*::*GALl-HIS3 GAL2-ADE2 met2*::*GAL7-lacZ* pGADC1 pGBDUC1	This study
PMY8	*MAT***a** *trpl-901 leu2-3,112 ura3-52 his3-200 ga14*Δ *ga180*Δ *LYS2*::*GALl-HIS3 GAL2-ADE2 met2*::*GAL7-lacZ* pGADC1*/RAD51* pGBDUC1*/RAD51*	This study
PMY9	*MAT***a** *trpl-901 leu2-3,112 ura3-52 his3-200 ga14*Δ *ga180*Δ *LYS2*::*GALl-HIS3 GAL2-ADE2 met2*::*GAL7-lacZ* pGADC1*/RAD51* pGBDUC1*/RAD52*	This study
PMY10	*MAT***a** *trpl-901 leu2-3,112 ura3-52 his3-200 ga14*Δ *ga180*Δ *LYS2*::*GALl-HIS3 GAL2-ADE2 met2*::*GAL7-lacZ* pGADC1*/ScRAD51* pGBDUC1*/RAD54*	This study
TSY10	*MAT***a** *trpl-901 leu2-3,112 ura3-52 his3-200 ga14*Δ *ga180*Δ *LYS2*::*GALl-HIS3 GAL2-ADE2 met2*::*GAL7-lacZ* pGADC1*/E108L-rad51* pGBDUC1*/E108L-rad51*	This study
PMY12	*MAT***a** *trpl-901 leu2-3,112 ura3-52 his3-200 ga14*Δ *ga180*Δ *LYS2*::*GALl-HIS3 GAL2-ADE2 met2*::*GAL7-lacZ* pGADC1*/E108L-rad51* pGBDUC1*/RAD52*	This study
PMY13	*MAT***a** *trpl-901 leu2-3,112 ura3-52 his3-200 ga14*Δ *ga180*Δ *LYS2*::*GALl-HIS3 GAL2-ADE2 met2*::*GAL7-lacZ* pGADC1*/E108L-rad51* pGBDUC1*/RAD54*	This study
PMY4	*MAT***a** *trpl-901 leu2-3,112 ura3-52 his3-200 ga14*Δ *ga180*Δ *LYS2*::*GALl-HIS3 GAL2-ADE2 met2*::*GAL7-lacZ* pGADC1 pGBDUC1*/RAD51*	This study
PMY7	*MAT***a** *trpl-901 leu2-3,112 ura3-52 his3-200 ga14*Δ *ga180*Δ *LYS2*::*GALl-HIS3 GAL2-ADE2 met2*::*GAL7-lacZ* pGADC1*/RAD51* pGBDUC1	This study
PMY14	*MAT***a** *trpl-901 leu2-3,112 ura3-52 his3-200 ga14*Δ *ga180*Δ *LYS2*::*GALl-HIS3 GAL2-ADE2 met2*::*GAL7-lacZ* pGADC1 pGBDUC1*/E108L-rad51*	This study
PMY11	*MAT***a** *trpl-901 leu2-3,112 ura3-52 his3-200 ga14*Δ *ga180*Δ *LYS2*::*GALl-HIS3 GAL2-ADE2 met2*::*GAL7-lacZ* pGADC1*/E108L-rad51* pGBDUC1	This study

### Yeast two-hybrid analysis.

Yeast two hybrid analysis was performed as described earlier (20). The strains PMY3, PMY8, PMY9, PMY10, TSY10, PMY12, PMY13, PMY4, PMY7, PMY14, and PMY11 were grown in SC-Ura-Leu medium until logarithmic phase. They were then diluted serially as shown in Fig. 5 and spotted on SC-uracil (Ura)-Leu and SC-Ura-Leu-adenine (Ade) medium. The plates were kept at 30°C for 3 to 4 days. The strain PMY3 was used as the negative control in our study.

### MMS sensitivity assay.

NRY1, NRY2, and TSY17 were tested for DNA damage sensitivity. All strains were grown in tryptophan dropout synthetic medium overnight at 30°C. The next day, a secondary culture was grown to an optical density at 600 nm (OD_600_) of 0.5 at 30°C. The culture was then divided into two sets. One set of cells was treated with 0.03% (vol/vol) of methyl methanesulfonate (MMS) (Sigma-Aldrich) and grown at 30°C for 2 h, and the other set was continuously grown at 30°C for 2 h without MMS. After that, the cells were serially diluted as mentioned, spotted on selective medium, and incubated at 30°C for 2 to 3 days.

### Gene conversion assay.

TSY20, TSY21, and TSY22 strains were generated by transforming pTA (empty vector), pTA-*RAD51* and pTA-*E108L-rad51*, respectively, into the NA14 Δ*rad51* strain. The transformed cells were initially patched on a plate containing glycerol as a sole carbon source. Next, equal numbers of cells were counted and spread on two different plates, one containing glycerol and other containing galactose as a carbon source, and incubated at 30°C for 3 to 5 days. Cells which survived on galactose plates were then patched on another plate containing G418 sulfate and incubated at 30°C for 36 h in order to determine the percentage gene conversion. Cells grown on G418 sulfate-containing plates utilize the Rad51-mediated gene conversion pathway for repair as they retain *KANMX6*. The ratio of the number of cells grown on the G418 sulfate plate to the number of cells grown on the galactose plate was calculated to determine the percent gene conversion. The assay was performed more than 3 times, and the mean values were plotted using GraphPad Prism.

### Chromatin immunoprecipitation.

TSY21 and TSY22 were grown in the selective medium to an OD_600_ of 0.3 in the presence of 3% glycerol. Half of the batch of cells was then treated with 3% galactose for 3 h, and other half continued to grow in glycerol medium. The ChIP assay was performed as described earlier (38). One microgram anti-Rad51 antibody was added to the sample to precipitate Rad51-bound DNA fragments. Recruitment of Rad51 was then monitored by PCR with 30 cycles using primer set OSB278/OSB279 in a reaction mixture volume of 50 µl using the immunoprecipitate and input DNA samples. Samples were subjected to electrophoresis on 2% agarose. For control, ChIP was performed with rabbit IgG antibody. To verify whether a double-stranded break (DSB) was generated by HO digestion in the assay strain, we used OSB289 as a forward primer, which is complementary to the 20 bp upstream of HO cut site (HOcs), and a reverse primer (KanB1) which is complementary to the *KANMX* gene. We amplified full-length *ACT1* using OSB14 and OSB16 as a normalization control.

### Western blotting.

Western blottin was performed to check Rad51 levels in NRY1, NRY2, and TSY17 strains. Protein samples were loaded on an SDS polyacrylamide gel. A polyvinylidene difluoride (PVDF) membrane was used for the transfer as described earlier (39). The primary antibodies used were mouse anti-Act1 (Abcam), rabbit anti-Rad51 (Santa Cruz), and mouse anti-Hsp82 (Calbiochem) at 1:5,000 dilutions. For subcellular fractionation, we used anti-Pgk1 antibody (Novus Biologicals) and mouse anti-Nsp1 antibody (Abcam) at 1:3,000 and 1:5,000 dilutions, respectively. For secondary antibodies, horseradish peroxide-conjugated anti-rabbit antibody (Promega) and anti-mouse antibody (Santa Cruz Biotechnology Inc., CA, USA) were used at 1:10,000 dilutions. The Western blots were developed using a chemiluminescent detection system (Pierce). Every experiment was repeated at least 3 times, and band intensities were quantified by using Image J software. Mean relative densities were plotted using GraphPad prism.

### Protein-protein docking.

The protein sequence of Rad51 with entry P25454 and the ATP-dependent molecular chaperone yHsp90 (Hsp82) with entry P02829 of Saccharomyces cerevisiae (strain ATCC 204508/S288c) are publicly available from the central repository of protein sequence and function, UniProt (Universal Protein Resource). The three-dimensional (3D) structures of Rad51 (PDB ID 1SZP) and yHsp90 (PDB ID 2CG9) were retrieved from the RCSB protein data bank. Protein-protein docking was conducted using a fully automated web-based program ClusPro 2.0, which employs an improved fast Fourier transform (FFT)-based rigid docking program PIPER. The program output is a short list of putative complexes ranked according to their clustering properties (18). Biovia Discovery Studio Visualizer is utilized for visualization and analysis of protein complexes. For mutation studies, the sequence of Rad51 protein retrieved from PDB (1SZP ABCDEF) was viewed in the sequence viewer of Biovia Discovery Studio software. The critical amino acids to be mutated were selected in all six chains and replaced. The sulfate ions were removed, and the structure of the protein generated was subjected to clean geometry and energy minimization before using for protein-protein docking. The amino acid Glu108 (E108) was mutated with four different amino acids, namely, leucine (E108L), alanine (E108A), glycine (E108G), and isoleucine (E108I), in chains A, B, C, D, E, and F to generate single mutant hexamers. The mutated Rad51 proteins were again subjected to protein-protein interaction with yHsp90 2CG9A. Protein-protein docking similar to that of the wild type was repeated with the mutant protein against Hsp90 using the online tool ClusPro.

### Coimmunoprecipitation.

Wild-type and *E108L-rad51* cells harboring yHsp90 overexpression plasmid (under GPD promoter; 2µ vector) were grown to an OD_600_ of 0.5. Ten milliliters of each culture was harvested, resuspended in 1 ml spheroplast buffer (50 mM Tris-HCl [pH 8], 25 mM HEPES [pH 7.4], 0.2% Casamino Acids, 0.2% yeast nitrogen base [YNB], 1% glucose, 18.2% sorbitol) containing dithiothreitol (DTT) and lyticase, and incubated at 30°C for 90 min. Subsequently, glass beads were added and the cells were intermittently vortexed and incubated on ice six times for a period of 30 s each. An anti-Rad51 antibody was added to the supernatant for overnight incubation at 4°C. Protein A agarose (25%; Calbiochem) was added, and the mixture was incubated for 2 h at room temperature. The beads were then spun down for 15 s at 1,000 rpm, and the pellet was washed 3 times with NETNS buffer (20 mM Tris-HCl [pH 8], 1 mM EDTA, 1 M NaCl, 0.5% [vol/vol] NP-40 with protease inhibitor) and twice with NETN buffer (20 mM Tris-HCl [pH 8], 1 mM EDTA, 100 mM NaCl, 0.5% [vol/vol] NP-40 with protease inhibitor). The bound protein was eluted with 4× Laemmli buffer by boiling for 10 min and was further spun down, and the supernatant was collected and used for Western blotting. The proteins in the supernatant were precipitated using 20% trichloroacetic acid, eluted using 4 × SDS loading dye containing dithiothreitol (DTT) and Tris (pH 8.8), and boiled for 10 min. The sample was spun down and the proteins in the supernatant were used for Western blotting. After the coimmunoprecipitation, the relative association of Hsp90 with Rad51 was calculated for each experiment using the following formula: relative association of Hsp90 with Rad51 = (Hsp90 in the pellet/Hsp90 in the input) ÷ (Rad51 in the pellet/Rad51 in the input).
